# Monthly Summary

**Published:** 1885-09

**Authors:** 


### Monthly Summary.

---

FRACTURE OF JAW AND TREATMENT.—Dr. Frank Abbott, New York, says: A lady patient, who was in the country during the summer, had the misfortune to be thrown from a carriage, striking on the right side of the lower jaw, in the region of the canine tooth. The jaw was thrown to the left, and, to all appearances, had a fracture near the angle on the left side. There was a fracture between the canine and lateral incisor on the right side. She was taken to a friend's house, and a surgeon—quite an eminent gentleman in Philadelphia—was telegraphed to come and attend her. He came, and did, I presume, what a good many surgeons would have done, viz., bound her head up with bandages as tightly as he could, tied her lower jaw in its place as nearly as he could, and left matters to take care of themselves. At the end of five weeks he took the bandage off and told her she was as well as she ever would be. She stayed at her friend's house some two weeks longer, then came home, and immediately came to my office and made an appointment. This was about the first of September. I saw her at the time appointed, and on examination discovered that the fracture on the right side had not healed. The pieces were loose. If there had been a fracture on the left side near the angle, as I concluded there had been—for I could account for the one-sided appearance of the lower part of the face in no other way—it had healed apparently as firmly as it ever was. On account of the moving of the parts she was unable to masticate, and consequently was unable to take anything in the way of solid food. After carefully looking the case over, and consulting with a surgeon in this city in reference to her physical condition, it was decided that the best plan to follow was to return the parts to as nearly their normal position as possible, by the easiest and most careful means possible, and retain them in position till they had united.

That was the treatment adopted. The operation consisted in taking an impression of the lower jaw, as perfect as we could get it, making a cast and set of dies, and striking up a gold plate. This was used instead of rubber for two reasons: First, because it is a conductor of heat and cold. Second, it could be placed in position more easily. Across the anterior part of the mouth a jack-screw was fitted, and a cut made in the plate, so that a portion of it might be moved independently of the rest. The question now was, how to hold them there and prevent the right side from tipping out to far. Fortunately a molar was missing on one side and a bicuspid on the other. Through these openings I passed a piece of wire, the ends of which were soldered to the plate. This was bent in the form of a hook. The apparatus was now ready and was placed in the mouth. By turning the jack-screw a very little each day for four or five days the parts were adjusted. The ends of the wire were then fastened around the outside of the teeth by means of a piece of small platinum wire, and thus the parts were retained immovably for five weeks. When the apparatus was removed, to the great delight of the patient, it was found, that a firm union of the fractured ends had taken place. By grinding off prominent points of the molars and bicuspids and forming inclined planes, the jaw was gradually worked around to the right, so that something approaching the original "bite" was obtained.

During the time the patient was wearing the apparatus she very faithfully rinsed her mouth six to ten times a day with antacids and anti-fermentative washes; consequently the teeth suffered very little.—*Proceedings of Odon. Society of New York, Cosmos.*

---

THE INTERNATIONAL MEDICAL CONGRESS.—The new Congress Committee of the American Medical Association met in Chicago on the 24th ult. Acting on legal advice, the original committee of seven decided to go to Chicago and act with the newly-appointed members. Several of the ablest lawyers in the country gave opinions to the effect that the American Medical Association possessed full control and power in the matter. The upshot of the meeting is a lively row. The chief change made in the sections was the removal of all new-coders from office, thereby displacing, among others, the chairmen of sections on Diseases of Children and on Laryngology—Drs. Jacobi and Lefferts of New York. Dr. H. I. Bowditch, of Boston, on account of alleged new code sympathies, was also removed from the office of Vice-President of the Congress. The membership of the Congress was limited to delegates from the American Medical Association and societies in affiliation with it. A complete change was made in the Executive Committee. Dr. Billings resigned the office of Secretary-General, and Dr. Packard, of Philadelphia, was nominated for the office. No sooner were the proceedings of the Chicago meeting made known than the members of the profession in the eastern cities interested in the organization met and expressed their emphatic disapproval of the action by unanimously refusing to have anything to do with the Congress under the present *regime*. We happened to be in Philadelphia on the occasion of the meeting in that city, and found that the leaders of the profession were of one mind in their determination to withdraw. Similar resolutions have been passed in Boston and Baltimore, and we may say that the majority of the men eminent in scientific medicine and surgery in the United States have decided to hold aloof from the Congress. When we ask why they take this serious step, we learn that they have, in the first place, a deep distrust of the American Medical Association as an organization which could satisfactorily carry out such an undertaking, and they have a still deeper distrust of the success of any congress where the best known scientific medical men of the country have been replaced by nominees such as Drs. Cole, Shoemaker, and others. The exclusion of the new-code men is felt to be a serious mistake, as carrying the quarrel of the American Medical Association into the International Medical Congress and refusing the fellowship of men with whom old-coders in New York are glad enough to consult. All, too, resent the insult offered to such a veteran as Henry I. Bowditch, of Boston, who has devoted half a century to the advancement of the best interests of the profession in the United States.

What the result will be it is difficult to predict, but it is hard to see how a Congress can be held without such men as those who have signified their intention of resigning. It would be like a in Congress in London without Paget, Lister, Jenner, Gull, Hutchinson, &c.; and we fear that when the profession in Europe hears of these dissensions, and the withdrawal of the very men they would be most anxious to meet, many who otherwise would have come will elect to stay away. —*Canada Med. and Surg. Journal.*

---

SAVING ROOTS.—Dr. J. Taft says: I can remember, and it is not very far back, that if there was a pulpless or an inflamed root in a mouth, the best method was thought to be to take it away, for fear it might do mischief at some future time; and often teeth were removed with the pulps alive. I have known many cases where good teeth were removed to make way for artificial dentures. Such would not be the treatment now. All teeth and roots that can be made useful in any way should be retained. They preserve the form of the features, and do good service. In a quarter of a century the practice has so changed that the removal of what was formerly regarded as a nuisance would now be severely censured. The subject is receiving as much attention as any now before the profession. The practice of some is commendable in this respect. They act like the skillful surgeon who endeavors to save any part that can be restored to usefulness. I belive the time will come when roots, such as are now destroyed by some of the most skillful, will be saved. If roots are allowed to remain they must, of course, be restored to health.

There are many methods of crowning these roots, though none of them are good for all cases. Only by wide study and conscientious efforts will you succeed. If you fail do not give up, but try again. We cannot expect to succeed at once with a new method, though it may have great merit. Sometimes a root will be rebellious, and we fail, but that should not discourage us. Physicians lose cases, so shall we be unsuccessful; we should go on in the expectation of success, and be exceedingly careful how we sacrifice teeth or roots that might be saved. If a pulp dies it must be without any assistance from me. A physician might as well decide to kill his patient because he thought he might not get well. Never use so deleterious an agent as arsenious acid.—*Items of Interest.*

---

ANCIENT TEETH.—A correspondent, says *Nature*, recently referred to the use of artificial teeth by the ancient Romans, as shown by a passage from Cicero, where one of the laws of the Twelve Tables is quoted. The law in question belongs to the Tenth Table (*de jure sacro*), which deals mainly with funerals, with the object of limiting the display and ceremonies attending them: Thus the body must not be burnt in more than three robes, or be attended to the grave by more than ten musicians; women must not tear their faces in time of mourning, nor must the bones be collected to make a new funeral with them, the bodies of slaves could not be embalmed, and the like. Section IX. of Table X., which is the one relating to teeth, reads as follows in Ortolan's text, ('Historie de la Legislation Romaine,' p. 121): "*Neve aurum addito. Quoi auro dentes vincti escunt, ast im cum illo sepelire urevere se fraude esto*"—Add no gold; but if the teeth are bound with gold, then that gold may be buried or burnt with the corpse. The date of the Twelve Tables is put about 450 B. C., and it is thought possible by some writers that some of the provisions relating to funerals were taken from the laws of Solon. It would, therefore, appear that dentistry was known and practised to some extent in the earliest period of their history by the Romans—to an extent, at any rate, that they used gold for binding the teeth. How the artificial teeth were made, or whether they had artificial teeth at all, is not apparent. In the case of the Estruscan skull mentioned recently in *Nature*, the teeth are made from the teeth of animals.—*East. Med. Journal.*

---

THE MOST POPULAR MEDICINES.—Last month we published a table compiled by an American writer showing the comparative proportion in which twelve of the leading medicines had been ordered in 1,000 prescriptions which had been taken at random. It was shown that quinine was ordered 238 times, opium 136, nux vomica 130, iron 123, while iodine, mercury, bismuth and bromine are altogether at 59 and 60 times. In June 1868, we published an article by Mr. W. Willmot on "Medicine," in which a somewhat similar investigation is recorded. Mr. Willmot had analysed 1,000 prescriptions, but he did not give the details in a form which admits of exact comparison. He, however, found that quinine was far ahead of any other single medicine ordered, but, classifying all remedies in their natural groups, he found mercury prominently at the top, then potash, bark, opium and iron.

He found that out of the 768 simple and compound mendicants of the Pharmacopœia, only 485 occurred at all in these 1,000 prescriptions, while three-fourths of these were not prescribed 10 times in the 1,000.—*Chemist and Druggist.*

---

CREOSOTE IN DENTITION.—During the last two years, I have been putting Creosote to a use that is, perhaps, new to some of your readers. I use it during the teething process of Infants. With febrile excitement combine with it aconite or any other indicated sedative, and if diarrhœa, etc., be present use Ipecac or Baptısta. I generally order Creosote x′, gtts. ii. to ıv., Water ℥ iv., M. Sig.: Teaspoonful two hours, later three to four hours. The Creosote x′. may be prepared by adding gtts. x, (ten drops) to Alcohol 98 per cent., 100 gtts. (100 dı ops). The practitioner who adds the above remedy to his pocket case and prescribes in the condition named, will certainly receive thanks from the mother of the little one for the promptness with which it has been relieved. —*C. M. Brucker, M. D., in Eastern Med. Journal.*

---

FOR INDIGESTION.—The season of the year is now upon us when the children begin to suffer from indigestion and *Cholera Infantum.* Light clothing and fresh air will do much toward allaying the irritable condition of the nervous system, regulated diet will help still more while such aids to digestion as *Lactopeptine* may be resorted to for lessening the task imposed on stomach and bowels. By such gentle and natural means good digestion may be coaxed back—surely a better treatment than the routine of opiates and astringents.—*Louisville Medical News.*

---

AN OLD BULLET.—J. B. Fanning, a resident of Newton county, Miss., in 1863 received a bullet wound ın the face during the progress of a battle. At the time of wounding it was thought best not to attempt to remove the ball. Last week it dropped into his mouth, having taken twenty-two years to work its way through.

REFLEX PAIN.—Dr. Black says: "With regard to this misplaced reference of pain or reflex pain as it is called, did you ever know such a thing as reflex pain when the sense of touch was implicated in the impressions? Touch is the great localizer in the nervous system; it has no other. When touch is involved the central cells have no record of an impression. There is no pain in the liver, or the cornea. The tooth pulp has no sense of touch and hence is incapable of localizing pain. The sense of touch, is requisite for the localization of pain in any part of the body. Pain in the knee in hip disease is an illustration. The pulp of the nerves has the power of transmitting sensations of pain only, and is utterly incapable of distinguishing between heat and cold, as I have frequently demonstrated. The peridental membrane has the sense of touch. Reflex actions is not within the control of the will.—*Items of Interest.*

