# Correction

**Published:** 1885-12

**Authors:** 


CORRECTION.—We have inadvertently published in the Sept. No. of this JOURNAL two selections which were credited to the *Items of Interest* but which, we are informed by Dr. Spalding, Editor of the *Archives of Dentistry* should have been credited to the latter Journal. We gladly make this correction as we were not aware of the claims of the *Archives of Dentistry* until so informed by its Editor, nothing appearing in the *Items of Interest* to indicate the ownership of the selections in question.

